# The inappropriate use of antibiotics in hospitalized dengue virus-infected children with presumed concurrent bacterial infection in teaching and private hospitals in Bandung, Indonesia

**DOI:** 10.1371/journal.pntd.0007438

**Published:** 2019-06-21

**Authors:** Riyadi Adrizain, Djatnika Setiabudi, Alex Chairulfatah

**Affiliations:** Department of Child’s Health, Faculty of Medicine Universitas Padjadjaran/Dr. Hasan Sadikin General Hospital, Bandung, Indonesia; University of Washington, UNITED STATES

## Abstract

**Background:**

Dengue virus infection (DVI) among children is a leading cause of hospitalization in endemic areas. Hospitalized patients are at risk of receiving unnecessary antibiotics.

**Methods:**

A retrospective medical review analysis study was conducted to evaluate the prevalence, indication, and choice of antibiotics given to hospitalized patients less than 15 years of age with DVI in two different hospital settings (teaching and private hospitals) in the Municipality of Bandung. Epidemiological, clinical, and laboratory data were obtained using a pre-tested standardized questionnaire from patients’ medical records admitted from January 1 to December 31, 2015.

**Results:**

There were 537 (17.5%) out of 3078 cases who received antibiotics. Among 176 cases admitted to the teaching hospitals, presumed bacterial upper respiratory tract infection (URTI) and typhoid fever were found in 1 (0.6%) case and 6 (0.3%) cases. In private hospitals among 2902 cases, presumed bacterial URTI was found in 324 (11.2%) cases, typhoid fever in 188 (6.5%) cases and urinary tract infection (UTI) in 18 (0.6%) cases. The prevalence of URTI and typhoid fever were significantly lower in the teaching hospitals compared to the private hospitals (p<0.0001 and p<0.05 respectively). The diagnosis of URTI in both teaching and private hospitals was merely based on clinical findings. Amoxicillin was given to 1 patient in the teaching hospitals; the 3^rd^ generation of cephalosporins, mostly intravenous, were given in 247 (67%) cases in private hospitals. The diagnosis of typhoid fever in the teaching hospitals was based on culture in 1 (16.7%) and reactive IgM anti-Salmonella in 5 (83.3%) cases while in the private hospitals, they were based on reactive IgM anti-Salmonella in 13 (6.5%) cases, single Widal test in 61 (32.5%), and without laboratory confirmation in 114 (60.6%) cases. Most of the cases in both hospital settings were treated mostly with 3^rd^ generation cephalosporin. The diagnosis of UTI was based on positive leucocyte esterase and nitrite in urine dipstick test in 7 (38.9%) and leucocyturia alone in 11 (61.1%) cases and was treated with 3^rd^ generation in 15 (83.3%) cases, amoxicillin, chloramphenicol and clarithromycin, each in 1 (5.6%) case.

**Conclusion:**

The use of antibiotics in private hospitals was inappropriate in most cases while the use of antibiotics in the teaching hospital was more accountable. This study indicated that interventions, such as the implementation of the antibiotics stewardship program, are needed especially in private hospitals to reduce inappropriate use of antibiotics.

## Author summary

DVI among children is a leading cause of hospitalization in endemic areas. In developing or underdeveloped countries, the antibiotics stewardship policy has not been well implemented yet. Thus, the risk of unnecessary antibiotics used in hospitalized patients becomes higher. Changing the way medical doctors use antibiotics is needed to greatly slow down the development and spread of antibiotic-resistant bacteria. We evaluate whether antibiotics administration given to hospitalized children with DVIinfection in two different hospital settings, teaching and private hospitals, were appropriate. The use of antibiotics in private hospitals was inappropriate in most cases while the use of antibiotics in the teaching hospital was more accountable. This study indicated that intervention should be made, such as the implementation of the antibiotics stewardship program, especially in private hospitals, to reduce inappropriate use of antibiotics.

## Introduction

The municipality of Bandung, like many other cities in Indonesia, is endemic for DVI. Indonesia belongs to the highest category (category A) for the endemicity of Dengue Fever (DF)/Dengue Haemorrhagic Fever (DHF) [1]. The Ministry of Health in 2016 reported [2], that DVI was found in all 34 provinces, and in 436 of 514 cities (85%) of Indonesia. In 2015, there were 126,675 reported cases, with 1229 fatality cases which were higher than in 2014 with 907 fatal cases among 100,347 reported cases.

The clinical manifestations of DVI range from mild illness to severe and sometimes fatal illness. The unpredictable outcome of DVI leads both parents and physicians to hospitalize patients, especially children. Hospitalized patients are prone to unnecessary antibiotics. In developing countries, the antibiotics stewardship policy has not been well implemented yet. Therefore, the risk of unnecessary antibiotics used in hospitalized patients becomes higher. Studies have shown that treatment indication, choice of agent or duration of antibiotic therapy is incorrect in 30% to 50% of cases [3]. One study of hospitalized adult patients showed that 19% patients received antibiotics regiments that were not clinically indicated. Among these patients, 20% experienced adverse drug effects, including 7 cases of Clostridium difficile infection [4]. The inappropriate use of antibiotics has detrimental effects on patients, healthcare system, and society [5]. It contributes to adverse drug effects, the emergence of antibiotics resistance and the rising cost of health care. Antibiotics are the most common cause of emergency visit for adverse drug effect in children under 18 years of age [6]. Antibiotic-resistance pathogens are a global public health problem, and there is a growing concern about the over-prescription of antibiotics in Asian countries [7].

We assumed that some of the hospitalized children with DVI, in both teaching and private hospitals, would receive antibiotics. We hypothesized that in a teaching hospital, as an academic institution, the prevalence of patients receiving antibiotics would be lower than in private hospitals, and the indication, as well as the choice of antibiotic administration, would be more accountable. The purpose of this study was to evaluate the prevalence, indication and, choice of antibiotics administration given to hospitalized children with DVI in two different hospital settings, teaching and private hospitals.

## Materials and methods

### Study design and period

This study was part of Hospital-based Dengue Case Surveillance in the Municipality of Bandung, West Java, Indonesia in 2015. This retrospective medical review analysis study was conducted from medical records of one teaching hospital and six private hospitals in Bandung.

Dr. Hasan Sadikin Teaching Hospital (HSTH) is a public hospital serving as teaching hospital for Faculty of Medicine, Universitas Padjadjaran, and also a referral hospital or tertiary care pediatric hospital for the Province of West Java, including the Municipality of Bandung. The private hospitals chosen based on their statuses of secondary and tertiary care hospitals in Bandung were: St. Borromeous Hospital (SBH), Adventist Hospital (AH), St. Yusuf Hospital (SYH), Hermina Mother and Child Hospital Pasteur (HMCHP), Limijati Mother and Child Hospital (LMCH), and Hermina Arcamanik Hospital (HAH).

### Study materials

Medical records of patients less than 15 years of age with DVI admitted to teaching and private hospitals in Bandung municipality from 1 January to 31 December 2015 were reviewed. Laboratory confirmation of DVI cases was the detection of NS-1 Dengue Antigen or serology test for the detection of IgM anti-Dengue. Cases with the unique clinical features of DHF and DSS, i.e. acute high fever followed by thrombocytopenia and the rising hematocrit value more than 20% with or without shock, even without laboratory confirmation, were considered as dengue infection cases [8]. Only medical records fulfilling the above criteria were included in this study.

In cases where patients received antibiotics during hospitalization, a thorough medical record review was then subsequently performed to evaluate the indication and the choice of antibiotics.

### Data collection tool

Using a pre-tested standardized questionnaire, the medical records of all DVI patients admitted from 1 January to 31 December 2015 were reviewed to obtain recorded epidemiological, clinical, and laboratory data.

### Study variables

The prevalence, indication in term of the diagnosis, and choice of antibiotics given to hospitalized children less than 15 years of age with DVI are the study variables in this study.

### Operational definitions

Inappropriate use of antibiotics in this study refers to overuse, such as unnecessary antibiotic administration in viral infection, or misuse, such as broad-spectrum cephalosphorin 3^rd^ generation given for bacterial upper respiratory tract infection.

### Data quality management

The pre-test for the questionnaire was done in the medical records department in other hospitals in Bandung to ensure sufficient data on epidemiological, clinical and laboratory data could be obtained. Data collectors were given training and were supervised daily by the principal investigator to collect quality data.

### Data analysis procedure

The comparison between two settings of hospital, teaching and private hospitals, were made using Pearson’s chi-square. Significance was set at p≤0.05.

### Ethic statement

Ethical clearance was obtained from the Research Ethics Committee of Faculty of Medicine Universitas Padjadjaran. The committee provided ethical clearance approval with all components of study protocol submitted to Bandung Municipality Health Authority to obtain permission to conduct this study in hospitals within the jurisdiction of the Municipality of Bandung. All data analyzed were further anonymized.

## Results

From January 1 to December 2015, there were 3.078 hospitalized DVI cases in children less than 15 years of age. Among them, 176 (5.7%) cases were hospitalized in Dr. Hasan Sadikin Teaching Hospital and 2902 cases (94.3%) were in 6 private hospitals. The proportion of Dengue Hemorrhagic Fever and the most severe form of DVI, Dengue Shock Syndrome (DSS), were found significantly higher in teaching hospitals (p<0.005 and p<0.0001, Table 1).

**Table 1:** Distribution of DVI patients based on hospital type.

Diagnosis	Teaching hospital n (%)	Private hospitals n (%)	Total	p value
Viral syndrome	2 (0.1)	5 (0.2)	7	0,0085
Dengue fever	58 (33.4)	1211 (41.7)	1269	0.0193
Dengue Hemorrhagic Fever	74 (42.0)	1543 (53.2)	1617	0.0034
Dengue Shock Syndrome	42 (23.9)	143 (4.9)	185	<0.0001

P value: Pearson’s chi-square

There were 547 (17.8%) out of 3078 patients that received antibiotics. In 10 (1.82%) cases the indication was due to recurrent shock. In 537 (17.5%) patients the indications of antibiotic administration were presumed concurrent bacterial infection as follows: Upper Respiratory Tract Infection (URTI) in 325 (60,5%), Typhoid Fever in 194 (36.1%), and Urinary Tract Infection (UTI) in 18 (3.4%) patients (Table 2). The prevalence of presumed concurrent bacterial infections, especially URTI, was significantly lower in the teaching hospital (p<0.0001, Table 2).

**Table 2:** DVI with presumed concurrent bacterial infection.

Presumed concurrent infection	Teaching hospital (176 cases) n (%)	Private hospitals (2902 cases) N (%)	p-value
URTI	1 (0.6)	324 (11.2)	<0.0001
Typhoid fever	6 (3.4)	188 (6.5)	<0.005
UTI	0	18 (0.6)	0.803
Total	7(4.0%)	530 (18.3)	

URTI: Upper respiratory tract infection, UTI: Urinary tract infection

P-value: Pearson’s chi-square

According to The Guidelines of Diagnosis and Treatment of DVI in Children, published by The Working Group on Infectious and Tropical Pediatric-Indonesian Pediatric Society [9], patients with Dengue Shock Syndrome experiencing recurrent shock should be given antibiotics. Therefore, only patients without recurrent shock receiving antibiotics were analyzed for the indication and the choice of antibiotics. Among 537 (17.5%) patients receiving antibiotics, the indications were due to Upper Respiratory Tract Infection (URTI) in 325 (60.5%), Typhoid Fever in 194 (36.1%), and Urinary Tract Infection (UTI) in 18 (3,4%) cases. The proportion of URTI and typhoid fever were significantly higher in private hospitals compared to the teaching hospital (0.6% vs. 11.2% and 3.4% vs. 6.5%, p<0.0001 and p<0.005 respectively, Table 2).

### DVI with presumed concurrent URTI

URTI as concurrent illness along with DVI was found in 325 cases (11.8%) of the total 3078 cases, or about 60.5% of 537 patients receiving antibiotics. The diagnosis of URTI in this study was merely based on clinical findings such as fever, sore throat, headache, cough, and redness of pharynx and or tonsils. Neither the Rapid Antigen Detection Test (RADT) nor throat culture is the practice in the diagnosis of URTI in Indonesia. One patient diagnosed as having concurrent URTI in the teaching hospital was 7 years old. The age distribution of URTI as the concurrent illness along with DVI in private hospitals is shown in Table 3.

**Table 3:** The age distribution of DVI with presumed URTI in private hospitals.

Age	n (%)
< 3 years of age	90 (27.8)
3–5 years of age	62 (19.1)
>5 years of age	172 (53.1)
**Total**	**324**

One patient (100%) in the teaching hospital was treated witj amoxicillin while patients in private hospitals receiving various antibiotics ranging from narrow to broad-spectrum antibiotics as shown in Table 4. The use of third generation cephalosporin, either intravenous or oral was found in 217 (67.%) cases as follows: cefixime oral in 87 (26.9%), ceftriaxone intravenous in 67 (20.7%), cefotaxime intravenous in 63 (19.4%) cases. Amoxicillin was used only in 6 (1.9%) cases.

**Table 4:** Antibiotics used in DVI with concurrent presumed URTI in private hospitals.

Antibiotics	n (%)
Aminopenicillins:	
Ampicillin oral	10 (3.1)
Amoxicillin oral	6 (1.9)
First generation cephalosporins:	
Cefadroxil oral	60 (18.5)
Cephalexin oral	1 (0.3)
Second generation cephalosporin:	
Cefuroxime i.v	2 (0.6)
Third generation cephalosporins:	
Ceftriaxone i.v	67 (20.7
Cefotaxime i.v	63 (19.4)
Cefixime (oral)	87 (26.9)
Others (quinolone, macrolides, thiamphenicol, aminoglycosides, metronidazole)	28 (8.6)
**Total**	**324 (100)**

### DVI with presumed concurrent typhoid fever

Six (3.4%) of 176 patients hospitalized in the teaching hospital with presumed concurrent typhoid fever underwent blood examination for culture and for detection of the IgM anti-Salmonella serology assay. The positive results were 16.7% and 83.3% respectively. In patients tested with positive for S. typhii isolation, the IgM anti-salmonella was undetected. No patient in this group underwent a single Widal test (Table 5).

**Table 5:** Laboratory confirmations among presumed concurrent typhoid fever.

	Teaching Hospital		Private Hospital		P value for (+) result
	n (%)	+result	n (%)	+result	
Blood Culture	6 (100)	1 (16.7)	None		N.A
IgM anti-Salmonella	6 (100)	5 (83.3)	106 (56.4)	13 (12.3)	<0.0001
Single Widal Test	None		65 (34.6)	61 (94.4)	N.A
No laboratory examination	None		17 (9.0)		N.A

P value: chi-square p <0.05

None of the patients hospitalized in private hospitals underwent blood culture. In 106 (56.4%) cases patients underwent serology assay for the detection of IgM anti-Salmonella. And, in 65 (34.6%) cases patients underwent single Widal test. The positive results were 12.3% and 94.4% respectively. The proportion of the positive result in serology assay for the detection of IgM anti-Salmonella in the teaching hospital was significantly higher compared to the private hospitals (83.3% vs. 12.3%, p<0.0001, Table 5). Seventeen (0.5%) patients in private hospitals did not undergo any laboratory confirmation, for which reason was not found in the medical records. In summary, in the private hospitals the laboratory confirmation was found in 74 (39.4%) cases.

All of the patients in the teaching hospital received ceftriaxone. In private hospitals, the presumed concurrent typhoid fever patients were treated with various antibiotics as shown in Table 6.

**Table 6:** Antibiotics used in DVI with concurrent presumed typhoid fever in private hospitals.

Antibiotics	n (%)
Aminopenicillins:	
Ampicillin	5 (2.7)
First generation cephalosporins:	
Cefadroxil	4 (2.1)
Second generation cephalosporin:	
Cefuroxime	1 (0.5)
Third generation cephalosporins:	
Ceftriaxone	88 (46.8)
Cefotaxime	40 (21.3)
Cefixime (oral)	25 (14.4)
Chloramphenicol	20 (10.6)
Quinolone (levofloxacine and ciprofloxacine)	5 (2.7)
Total	188 (100)

### DVI with presumed concurrent urinary tract infection (UTI)

All of the 18 cases with presumed concurrent UTI were hospitalized in private hospitals. The diagnosis was based on positive nitrite and leukocyte esterase on dipstick test in 7 (38,9%) and leukocyturia alone in 11 (61.1%) cases. Cefotaxime was given in 6 (33.3%) cases, cefixime in 5 (27.8%), ceftriaxone in 4 (22.2%%); Amoxicillin, Chloramphenicol and clarithromycin each in 1 (5.6%) case.

## Discussion

There were 547 (17.8%) out of 3078 patients that received antibiotics. In 10 (1.82%) cases the indication was due to recurrent shock. In the Indonesian Guidelines of Diagnosis and Treatment of DVI in Children [9] recurrent shock is an indication for antibiotic administration due to the possibility of bacterial translocation. Therefore, a thorough medical record review to evaluate the indications and the choice of antibiotics were performed only in DVI patients without recurrent shock. The teaching hospital as an academic hospital had a more judicious policy in determining the diagnosis of presumed concurrent bacterial infection. This policy will decrease unnecessary antibiotic administration.

### DVI with presumed concurrent URTI

This study revealed that although clinical findings cannot differentiate between viral and bacterial URTI, all of the patients received antibiotics. The main reason for giving antibiotics is Group A β Streptococcal (GAS) pharyngitis to avoid rheumatic fever complication. However, most of URTI in children are of viral origin. GAS pharyngitis is very rare in children less than 3 years of age. In developed countries where RADT and culture are available, RADT should generally not be performed in children younger than 3 years, in whom GAS rarely causes pharyngitis and in whom rheumatic fever is uncommon [10]. In children less than 3 years, presumed concurrent URTI in private hospitals was found in 90 (22.8%) cases (Table 3), and all of them received antibiotics. Moreover, given that the prevalence of streptococcal infection among URTI patients in Asia-Pacific region is not more than 20% [11], the administration of antibiotics to all 324 (100%) patients with presumed URTI in this study is considered over-prescription. The signs and symptoms of some cases of DF/DHF can mimic URTI such as an injected pharynx, cough, and coryza [8] that will disappear as the illness subsides. The overuse of antibiotics has also been found in other studies where antibiotics for viral URTI has been studied with the rates of overuse as high as 13% to 75% [12–14]. Since oral penicillin V is not available in Indonesia, amoxicillin becomes the first line drug for treating GAS pharyngitis [10], in teaching hospital this antibiotic was given in 1 patient (100%) while it was given in only in 6 (1.9%) in private hospitals. Table 4 shows that all of presumed concurrent URTI patients in private hospitals received antibiotics, ranging from narrow spectrum to broad-spectrum of antibiotics, orally or intravenously. The broad-spectrum 3^rd^ generation cephalosporin was given in 217 (67%) cases, mostly ceftriaxone and cefotaxime (130 or 40.1% cases). The use of this agent for URTI is considered as misuse of antibiotics, which can result in detrimental effects for the patients, and the healthcare system, including the emergence of antibiotic resistant pathogens [5]. Antibiotics frequently associated with Clostridium difficile infection is 3^rd^ generation cephalosporin [6]. This study shows that quinolone was also given to URTI patients. This broad-spectrum antibiotic should be used judiciously, as resistant bacteria develop quickly. In the increased use of fluoroquinolone, the associated infection caused by fluoroquinolone-resistant, hypervirulent Clostridium difficile has been reported [15]. The prescribing of broad-spectrum antibiotics in pediatrics is extremely common and frequently inappropriate [16]. Despite the guidelines for group A pharyngitis diagnosis and treatment had been published and available, pediatricians over-treating and mistreating sore throats in children are still often found [17].

One patient received metronidazole for presumed concurrent URTI, this treatment is not indicated since such antibiotic has poor efficacy against aerobic gram-positive like streptococci spp., metronidazole’s spectrum of activity is limited to obligate anaerobic bacteria and some microaerophilic bacteria that normally thrive in the presence of the low concentration of oxygen [18]. We conclude that the indications and the choices of antibiotics in DVI with presumed concurrent URTI, especially in private hospital were inappropriate.

### DVI with presumed concurrent typhoid fever

Typhoid is an enteric fever caused by S. typhii. This illness is endemic in the Middle East, Africa, Central and South America, Indian Subcontinent, East Asia and in South East Asia including Indonesia [19]. The definitive diagnosis of typhoid fever requires isolation of the organism from blood or bone marrow. However, it takes time to obtain the result. For the purpose of early diagnosis and treatment, rapid serology diagnostic tests such as the Widal test and IgM anti-Salmonella rapid test are usually used while waiting for culture result. With the high prevalence of antibodies among healthy individuals in an endemic area, a single traditional Widal test lacks sensitivity to diagnose typhoid fever [19,20]. The teaching hospital did not use this test anymore, but it was still done in 65 (34.6%) cases in private hospitals. This study showed that among 65 cases undergoing a single Widal test there were positive results in 61 (94.4%) cases. The IgM anti-Salmonella rapid test is more specific in the diagnosis of typhoid fever, showing a sensitivity of 78–79% and specificity of 89% [21].

This study shows that blood specimens had been obtained from all patient in teaching hospital for culture and IgM detection serology assay. The diagnosis of typhoid fever in the teaching hospital was supported by culture and IgM anti-salmonella in one (16.7%) and in five (83.3%) cases respectively. The 106 cases in private hospitals were tested for IgM anti-salmonella detection, with the positive result found in 13 (12.3%) cases. It was significantly lower compared to the teaching hospital (83.3% and 12.3% p<0.0001, Table 5). The high percentage of reactive IgM anti-salmonella test in the teaching hospital lead to the assumption that clinical suspicion in the teaching hospital was more accurate than in the private hospitals. In summary, in private hospitals the laboratory confirmation case was found in 74 (39.4%) cases, consisting of reactive for IgM anti-Salmonella in 13 (6.9%) and single Widal test in 61 (32.5%). The other 114 (60.6%) cases were without laboratory confirmation.

Despite the inaccuracy in the diagnosis of typhoid fever in private hospitals, all patients received antibiotics as shown in Table 6. Chloramphenicol, an inexpensive and time-honored drug is still a first line in the treatment of typhoid fever in Indonesia despite a high prevalence of Salmonella resistant to chloramphenicol have been reported elsewhere, but no resistance strain was found fin Indonesia [22]. Although chloramphenicol continues to be used in countries without resistance to it, this old drug exposes patients to serious toxicity. The rare but often fatal side effect is bone marrow aplasia. The awareness of marrow aplasia, make this drug has been set aside especially in managing typhoid fever concurrently with dengue hemorrhagic fever, with typically has thrombocytopenia. This study shows that in the teaching hospital, all of the patients received ceftriaxone and none received chloramphenicol. In the private hospitals, chloramphenicol was given in 20 (10.6%) cases. Ceftriaxone alone was the most often used antibiotics given in 93 (47.9%) cases, followed by cefixime in 25 (12.9%), and cefotaxime in 20 (10.6%) cases (Table 6). Ceftriaxone in many trials did not exhibit marrow suppression and had a shorter duration of treatment as well as lower relapse rate [23–26]. In summary, the diagnosis of typhoid fever and the choice of antibiotics was accountable in the teaching hospital, but in most cases in private hospitals, the diagnosis of typhoid fever was without support of laboratory confirmation which could lead to unnecessary antibiotic prescription.

### DVI with presumed concurrent UTI

Presumed concurrent UTI in DVI cases were found in 18 cases in private hospitals. The diagnosis was based on positive nitrite and leucocyte esterase on the dipstick test in 7 (38,9%) and leucocyturia alone in 11 (61.1%) cases. The criteria for rapid UTI diagnosis based on the findings of the dipstick test and microscopy examination has been proposed by the National Institute for Health and Care Excellence (NICE) [27]. If both leukocyte esterase and nitrite are positive, the child should be regarded as having UTI and antibiotic treatment should be initiated. This study shows that only in 6 (33.3%) met the criteria of UTI above.

The Nephrology Working Group-Indonesian Pediatric Society [28], recommends ceftriaxone as the first line drug in the treatment of UTI. This study shows that cefotaxime, ceftriaxone, cefixime were given in 6 (33.3%), 5 (27.8%)) and 4 (22.2%) cases respectively. Amoxicillin, chloramphenicol, and clarithromycin, each was given in 1 (5.6%) case. Since the urinary concentration of chloramphenicol is low, this drug is not intended to treat UTI [29].

We conclude that the diagnosis of UTI in most cases was not sufficient, and the choice of antibiotics was inappropriate in some cases.

### Conclusion

The purpose of this study was to evaluate the prevalence, indication and choice of antibiotics administration given to hospitalized children with DVI in two different hospital settings, namely teaching and private hospitals. This study showed that the proportion of dengue haemorrhagic fever and the most severe DVI, dengue shock syndrome, were found significantly higher in the teaching hospital (p<0.005 and p<0.0001 respectively, Table 1). We conclude that both the indication and the choice of antibiotics in hospitalized DVI with the presumed concurrent bacterial infection in this study were inappropriate in most cases, especially in private hospitals. Perhaps, the single most important action needed to greatly slow down the development and spread of antibiotic-resistant bacteria is to change the way antibiotics are used [30].

### Limitation of the study

This study is a retrospective medical record study as we cannot obtain the unwritten reason in evaluating the indication and the choice of antibiotics administration. In private hospitals the laboratory confirmation was found in 74 (39.4%) cases. In terms of sampling and sample size, only one teaching hospital was used while 5 private hospitals were used. This could have affected the analysis and introduced bias, built into the study.

## Supporting information

S1 Checklist STROBE checklist. (DOC)
