# Supplementary material for: The inappropriate use of antibiotics in hospitalized dengue virus-infected children with presumed concurrent bacterial infection in teaching and private hospitals in Bandung, Indonesia
Source: PLoS Negl Trop Dis. 2019 Jun 21;13(6):e0007438. doi: 10.1371/journal.pntd.0007438 (PMC6608981; doi:10.1371/journal.pntd.0007438)
Supplement: S1 Checklist — (DOC) [file pntd.0007438.s001.doc]

STROBE Statement—Checklist of items that should be included in reports of ***cross-sectional studies***

|  | Item No | Recommendation |
| --- | --- | --- |
| **Title and abstract** | 1 | (*a*) Indicate the study’s design with a commonly used term in the title or the abstract  **Respond:** It is explained in the title page, page 1 line 6 |
|  |  | (*b*) Provide in the abstract an informative and balanced summary of what was done and what was found  **Respond:** It is explained in the abstract, page 2 line 2 |
| Introduction | | |
| Background/rationale | 2 | Explain the scientific background and rationale for the investigation being reported  **Respond:** It is explained in the introduction, page 4 line 14-17 |
| Objectives | 3 | State specific objectives, including any prespecified hypotheses  **Respond:** It is explained in the introduction, page 4 line 29 |
| Methods | | |
| Study design | 4 | Present key elements of study design early in the paper  **Respond:** It is explained in page 5 line 9-10 |
| Setting | 5 | Describe the setting, locations, and relevant dates, including periods of recruitment, exposure, follow-up, and data collection  **Respond:** It is explained in page 5, line 11-19 |
| Participants | 6 | (*a*) Give the eligibility criteria, and the sources and methods of selection of participants  **Respond:** It is explained in page 5, line 25-30 |
| Variables | 7 | Clearly define all outcomes, exposures, predictors, potential confounders, and effect modifiers. Give diagnostic criteria, if applicable  **Respond:** It is explained in page 6, line 10-13 |
| Data sources/ measurement | 8* | For each variable of interest, give sources of data and details of methods of assessment (measurement). Describe comparability of assessment methods if there is more than one group  **Respond:** It is explained in page 5, line 25-30 |
| Bias | 9 | Describe any efforts to address potential sources of bias  **Respond:** It is explained in page 5, line 21-24 |
| Study size | 10 | Explain how the study size was arrived at  **Respond:** It is explained in page 5, line 25-30 |
| Quantitative variables | 11 | Explain how quantitative variables were handled in the analyses. If applicable, describe which groupings were chosen and why  **Respond:** It is explained in page 6, line 28-29 |
| Statistical methods | 12 | (*a*) Describe all statistical methods, including those used to control for confounding  **Respond:** It is explained in page 6, line 28-29 |
|  |  | (*b*) Describe any methods used to examine subgroups and interactions  **Respond:** No subgroups and interaction needed to be described |
|  |  | (*c*) Explain how missing data were addressed  **Respond:** We used data from medical records, incomplete records were not submitted |
|  |  | (*d*) If applicable, describe analytical methods taking account of sampling strategy  **Respond:** We took all the samples available into account |
|  |  | (*e*) Describe any sensitivity analyses  **Respond:** We did not need any sensitivity analyses |
| Results | | |
| Participants | 13* | (a) Report numbers of individuals at each stage of study—eg numbers potentially eligible, examined for eligibility, confirmed eligible, included in the study, completing follow-up, and analysed  **Respond:** It is explained in page 5, line 11-19 |
|  |  | (b) Give reasons for non-participation at each stage  **Respond:** We did a medical record review – no participants. |
|  |  | (c) Consider use of a flow diagram |
| Descriptive data | 14* | (a) Give characteristics of study participants (eg demographic, clinical, social) and information on exposures and potential confounders  **Respond:** It is explained in Table 3 |
|  |  | (b) Indicate number of participants with missing data for each variable of interest  **Respond:** We included all data |
| Outcome data | 15* | Report numbers of outcome events or summary measures  **Respond:** It is explained in page 16 line 11 |
| Main results | 16 | (*a*) Give unadjusted estimates and, if applicable, confounder-adjusted estimates and their precision (eg, 95% confidence interval). Make clear which confounders were adjusted for and why they were included  **Respond:** It is explained in page 16, line 11 |
|  |  | (*b*) Report category boundaries when continuous variables were categorized  **Respond:** No categorized variables |
|  |  | (*c*) If relevant, consider translating estimates of relative risk into absolute risk for a meaningful time period |
| Other analyses | 17 | Report other analyses done—eg analyses of subgroups and interactions, and sensitivity analyses |
| Discussion | | |
| Key results | 18 | Summarise key results with reference to study objectives  **Respond:** It is explained in page 16, line 11 |
| Limitations | 19 | Discuss limitations of the study, taking into account sources of potential bias or imprecision. Discuss both direction and magnitude of any potential bias  **Respond:** It is explained in page 16, line 29 |
| Interpretation | 20 | Give a cautious overall interpretation of results considering objectives, limitations, multiplicity of analyses, results from similar studies, and other relevant evidence  **Respond:** It is explained in page 16, line 11 |
| Generalisability | 21 | Discuss the generalisability (external validity) of the study results  **Respond:** It is explained in page 16, line 11 |
| Other information | | |
| Funding | 22 | Give the source of funding and the role of the funders for the present study and, if applicable, for the original study on which the present article is based |

*Give information separately for exposed and unexposed groups.

**Note:** An Explanation and Elaboration article discusses each checklist item and gives methodological background and published examples of transparent reporting. The STROBE checklist is best used in conjunction with this article (freely available on the Web sites of PLoS Medicine at http://www.plosmedicine.org/, Annals of Internal Medicine at http://www.annals.org/, and Epidemiology at http://www.epidem.com/). Information on the STROBE Initiative is available at www.strobe-statement.org.
